# Processing and Characterization of Pendidam: A Skimmed Fermented Milk Locally Made in the Far North of Cameroon

**DOI:** 10.1155/ijfo/7743110

**Published:** 2026-07-04

**Authors:** James Ronald Bayoï, Simplice Noubasra

**Affiliations:** ^1^ Department of Biological Sciences, Faculty of Sciences, University of Maroua, Maroua, Far North Region, Cameroon, uni-maroua.citi.cm

**Keywords:** Cameroon, food safety, microbial contamination, nutritional composition, pendidam, traditional fermented cow milk

## Abstract

Pendidam is a traditional, skimmed fermented milk of cultural and dietary importance in the Far North Region of Cameroon. Despite being produced using rudimentary techniques, its quality and safety remain uncharacterized. This study is aimed at documenting its production process and evaluating its physicochemical, nutritional, microbiological, and sensory properties. We conducted surveys and group discussions with producers across the region and collected samples for analysis using standard methods. Pendidam production is a female‐dominated activity rooted in pastoral communities, involving predominantly married and uneducated producers with extensive experience acquired through traditional knowledge. The process relies on unstandardized artisanal practices and spontaneous fermentation that may pose contamination risks. Although pendidam milk exhibited a desirable acidic pH lower than 4 (from 3.35 to 3.66) and contained proteins (7.84–9.13 g/L), free amino acids (5.61–7.77 g/L), and sugars (7.68–8.87 g/L), its microbiological quality was unsatisfactory. High counts of fungi (5.48–5.78 Log_10_ CFU/mL), spore‐forming bacteria (4.44–4.90 Log_10_ CFU/mL), and staphylococci (2.79–2.99 Log_10_ CFU/mL) were recorded exceeding standard safety thresholds, and the sulfite‐reducing clostridia and coliforms were also detected. Sensory analysis revealed high consumer acceptability. Pendidam possesses substantial nutritional potential but is critically compromised by hygiene deficiencies from unhygienic practices. There is an urgent need for targeted interventions, including formal hygiene training and process optimization, to ensure its safety and support its valorization.

## 1. Introduction

Global milk production has shown sustained growth over the past decades, reaching approximately 965 billion liters in 2023 and nearly 985 billion liters in 2024, according to FAO estimates [1]. Although Africa contributes approximately 6% of this output [2], with Cameroon producing between 886 and 900 million liters, the Far North Region of Cameroon presents a unique paradox [3]. In this region, milk is deeply embedded in pastoral livelihoods and cultural traditions, yet its preservation remains highly constrained by extreme climatic conditions, where temperatures can reach up to 42°C and access to refrigeration systems is limited. These constraints significantly increase postharvest losses and reduce the availability of safe dairy products, particularly in rural communities. As a result, local populations, primarily women, have developed indigenous fermentation techniques to convert highly perishable raw milk into more stable products such as artisanal yoghurt, kindirmou, and pendidam [3]. These products are not only essential for food security and nutrition, but also constitute a key source of household income, especially for women engaged in informal dairy value chains.

Fermentation is one of the oldest and most effective methods of food preservation [4]. Through the metabolic activity of lactic acid bacteria (LAB), lactose is converted into lactic acid, lowering the pH and thereby inhibiting the growth of spoilage and pathogenic microorganisms while contributing to desirable sensory characteristics [5, 6]. This process is particularly important for raw milk, which, due to its rich composition in proteins, fats, lactose, vitamins, and minerals, provides an excellent substrate for microbial growth [7, 8]. However, while fermentation enhances shelf life and safety, the final quality of fermented dairy products remains highly dependent on processing conditions and hygiene practices [9].

In many parts of Africa, including the Far North Region of Cameroon, traditional fermented milks are produced using empirical knowledge passed down through generations [4], often without standardized hygiene measures, controlled fermentation parameters, or the use of defined starter cultures [10]. This artisanal context introduces substantial variability in product quality and increases the risk of contamination by undesirable microorganisms such as coliforms, staphylococci, yeasts, and molds. In regions where such products are consumed daily and often without further heat treatment, these microbiological risks represent a significant but underdocumented public health concern. [11, 12].

Among these traditional products, pendidam is a fermented dairy beverage made from raw cow′s milk, with preparation relying on rudimentary equipment and the specialized knowledge of local women [13]. This skimmed and churned fermented milk is widely consumed across all socioeconomic groups in the Far North Region [3]. Beyond its dietary role, pendidam plays a strategic socioeconomic function: it contributes to income generation for rural women, supports pastoral economies, and enhances the valorization of locally produced milk in a context of limited industrial processing infrastructure. Despite this importance, pendidam remains poorly documented in scientific literature. Although studies on similar African fermented milks such as raieb and leben in North Africa [14], nunu in Ghana [15], and kindirmou in Cameroon [16] have documented insights into their production and quality attributes, data on pendidam is scarce and fragmented. Existing studies have mainly focused on isolated aspects such as microbiological or compositional characteristics [11, 16, 17], without offering an integrated understanding of the product.

This lack of comprehensive scientific data has several concrete implications. First, it limits the ability to identify critical control points within the production process, thereby constraining efforts to improve hygiene and ensure consumer safety. Second, it hinders the development of standardized processing guidelines that could enhance product consistency and shelf life. Third, it restricts opportunities for valorization and scaling‐up of pendidam within formal markets, as quality assurance and regulatory compliance require well‐documented product characteristics. Finally, it weakens the capacity of policymakers and development actors to design targeted interventions that support local dairy value chains and strengthen food security in this climate‐vulnerable region.

This study, therefore, is aimed at providing the comprehensive characterization of pendidam by documenting its traditional production process and systematically evaluating its physicochemical, nutritional, microbiological, and sensory properties. By establishing a robust scientific reference framework, this work seeks to support evidence‐based strategies to improve product quality and safety, and promote the sustainable development and preservation of this culturally significant fermented milk.

## 2. Materials and Methods

### 2.1. Reagents and Chemicals

Sodium hydroxide (NaOH), ethanol (C_2_H_6_O), ammonium sulfate ((NH_4_)SO_4_), sodium acetate (C_2_H_3_O_2_Na), formaldehyde (CH_2_O), and acetylacetone (C_5_H_8_O_2_) were purchased from Sigma‐Aldrich (Mumbai, India). Glucose (C_6_H_12_O_6_), galactose (C_6_H_12_O_6_), alanine (C_3_H_7_NO_2_), and orcinol (C_7_H_8_O_2_) were obtained from Columbia Biosciences (United Kingdom). Ninhydrin (C_9_H_6_O_4_) and dinitrosalicylic acid (C_7_H_4_N_2_O_7_) were gifted by the Bioaliment TehnIA (Dunarea de Jos University of Galati, Romania). Reagents and chemicals requisite for this research were of analytical grade.

### 2.2. Study Area

**Figure 1:**
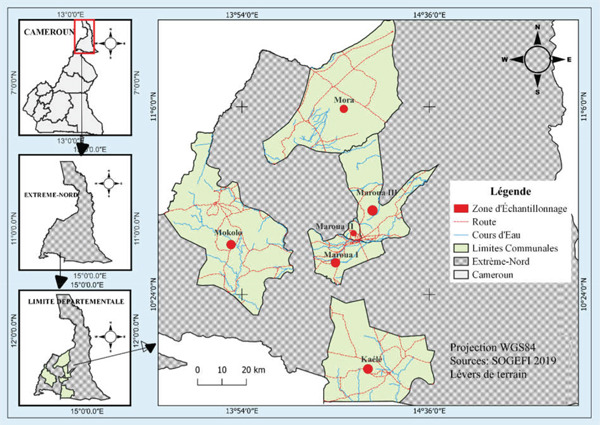
Map of survey and sampling sites.

This study was carried out in the Far North Cameroon that covers a total area of 34.262 km^2^ counting about for 10% of the national territory. The Far North Region, with approximately 3 million people, is the second most densely populated region in the country. This region, located in the northern part of the country, is bordered by Chad and Nigeria. Four study cities, including Maroua, Mora, Kaélé, and Mokolo, were selected based on the frequency of pendidam marketing and product availability (Figure 1).

### 2.3. Survey on Production Process and Sociodemographic Characteristics

A questionnaire‐based survey was conducted through face‐to‐face interviews using a structured questionnaire, complemented by group discussions, among 80 producers across the four study sites. The questionnaire collected information on sociocultural characteristics (gender, origin, age, marital status, family size, educational level, and production experience), sources of raw milk, detailed production steps, packaging methods, production volume, and storage practices. In addition, the production environment, vending conditions, and equipment used, including calabashes, pots, and other utensils, were visually inspected.

### 2.4. Sample Collection

A total of 24 pendidam samples were collected aseptically, including seven from Maroua, six each from Kaélé and Mora, and five from Mokolo. Samples were obtained from vendors using presterilized plastic containers, approximately 0.35 L of each sample was transferred and labeled. Immediately after collection, samples were stored in a refrigerated icebox and transported under cold chain conditions to the laboratory for analysis.

### 2.5. Physicochemical Analyses

All physicochemical analyses were performed in triplicate using international standardized procedures. The pH was measured directly with a calibrated portable pH meter (Eco Testr, Singapore) by immersing the electrode in 25 mL of sample. Titratable acidity (TTA), expressed as a percentage of lactic acid, was determined by titrating 25 mL of sample with 0.1‐N sodium hydroxide using phenolphthalein as an indicator until a stable pale pink color appeared [18].

Soluble solids (°Brix), total dissolved solids (TDS), and electrical conductivity (EC) were measured using a handheld refractometer (RHB 90, Shengzen, China) and a portable multifunction conductivity meter (e‐1 TDS&EC, Shengzen, China), respectively. Temperature, expressed in degrees Celsius (°C), was recorded using a hand pen thermometer (TP300, Shengzen, China).

Total dry matter (DM) was determined by drying in an air oven (Memmert GmbH, Germany) approximately 10 mL of sample at 105°C for 24 h until constant weight was obtained [19]. Ash content was measured by incinerating in a furnace (Nabertherm, Germany) about 2 mL of sample at 550°C for 6 h, followed by cooling in a desiccator and weighing [19]. Both previous parameters were expressed as percentage mass to mass (%, m/m).

### 2.6. Biochemical and Nutritional Analyses

Total sugar content was determined spectrophotometrically at 450 nm using the orcinol method, in which soluble sugars (SS) react with orcinol in a hot acidic medium to form a yellow–orange complex [20]. Sample (0.5 mL) or 0.5 mL of glucose (0–100 *μ*g/mL) and 1500 *μ*L of 0.1% orcinol were mixed and heated in water bath (Memmert GmbH, Germany) at 90°C for 15 min. After cooling at room temperature, the optical density was read at the wavelength of 450 nm using a UV–vis spectrophotometer (Jenway 7305, Bibby Scientifc, Staffordshire, United Kingdom), and the SS content was expressed as g/L from a standard calibration curve of glucose (*y* = 0.0312*x*–0.0247, *R* ^2^ = 0.99). Reducing sugars (RS) were quantified by the 3,5‐dinitrosalicylic acid (DNSA) method, based on the formation of a colored complex measured at 530 nm [21]. One milliliter of DNSA reagent was added to 0.5 mL of sample or galactose (0–25 *μ*g/mL), and the mixture was heated in water bath (Memmert GmbH, Germany) for 5 min. After cooling at room temperature, 1 mL of distilled water was added, and the optical density was read at 530 nm wavelength thanks to a UV–vis spectrophotometer (Jenway 7305, Bibby Scientifc, Staffordshire, United Kingdom). The RS was expressed as g/L using a standard curve of galactose (*y* = 0.0302*x*–0.0237; *R* ^2^ = 0.98).

Total proteins were assessed using a colorimetric method involving the reaction of nitrogen with acetylacetone and formaldehyde, resulting in the formation of a yellow complex (3,5‐diacetyl‐1,4‐dihydrolutidine) measured at 412 nm [22]. About 0.1 mL of sample or ammonium sulfate (0–10 *μ*g/mL) and 0.6 mL of sodium acetate (0.08 g/mL) and 0.8 mL of a reagent (composed of 15 mL of 37% formaldehyde, 7.8 mL of acetylacetone completed to 100 mL with distilled water) were mixed. The mixture was shaken for 5 min and kept in a water bath for 15 min at 90°C. After cooling, 3.5 mL of distilled water were added, the mixture was vortexed for 1 min, and the optical density was read at 412 wavelength using a UV–vis spectrophotometer (V‐1100d JP, Selecta, Spain). The nitrogen content was calculated using the standard curve of ammonium sulfate (*y* = 0.0502*x*–0.0137: *R* ^2^ = 0.98). The nitrogen was converted into protein content (expressed as g/L) using a conversion factor of 6.38. A nitrogen‐to‐protein conversion factor of 6.38 was used because it is the standard factor recommended for milk and dairy products, reflecting the specific nitrogen content of milk proteins more accurately than the general factor of 6.25. Free amino acids (AA) were evaluated using the ninhydrin method, in which AA react with ninhydrin at 90°C to produce a purple‐colored complex measured at 530 nm [23]. A volume of 0.1 mL of sample or standard alanine solution (0–250 *μ*g/mL) and 0.5 mL of 1% ninhydrin were mixed. After vigorous shaking, the mixture was heated at 90°C for 5 min in a water bath. The reaction mixture was cooled at room temperature; then, 0.5 mL of 50% ethanol was added, followed by reading of optical density at 530 nm wavelength using a UV–vis spectrophotometer (V‐1100d JP, Selecta, Spain). The standard calibration curve (*y* = 0.0294*x*–0.0085; *R* ^2^ = 0.99) of alanine was plotted and used for determination of free AA content, expressed as g/L.

### 2.7. Microbiological Analyses

Microbiological analyses were conducted using the standard decimal dilution technique in sterile physiological saline (0.85% NaCl), followed by surface plating on selective and differential media according to International Dairy Federation guidelines [24]. Total aerobic mesophilic flora (TAMF) was enumerated on plate count agar (PCA, Lioflchem, Teramo, Italy) incubated at 35°C for 24–48 h. Mesophilic spore‐forming bacteria (SFB) were determined after heat treatment of samples at 80°C for 10 min, followed by plating on PCA and incubation at 35°C for 48 h. Yeasts and molds (total fungi [TF]) were counted on potato dextrose agar (PDA, Lioflchem, Teramo, Italy) incubated at 25°C for 3–5 days. Total and fecal coliforms (TC and FC) were detected using Eosin Methylene Blue (EMB, Lioflchem, Teramo, Italy) agar incubated at 37°C, and Violet crystal and neutral Red Bile Lactose agar (VRBL, Condalab, Madrid, Spain) incubated at 44°C, respectively. Fecal streptococci (FS) were enumerated on Slanetz and Bartley agar (Oxoid, Hampshire, United Kingdom) incubated at 37°C for 48 h. Staphylococci (Staph) were isolated on mannitol salt agar (MSA, Lioflchem, Teramo, Italy) incubated at 37°C for 24 h. Sulfite‐reducing clostridia (RSC) were detected after heat treatment, followed by anaerobic incubation on trypticase sulfite neomycin (TSN, Condalab, Madrid, Spain) agar at 46°C for 48 h. Lactic acid bacteria (LAB) were enumerated on de Man–Rogosa–Sharpe agar (MRS, Lioflchem, Teramo, Italy) under anaerobic conditions at 37°C for 48 h. *Salmonella* and *Shigella* were investigated through pre‐enrichment in buffered peptone water, selective enrichment in selenite cystine broth, and isolation on *Salmonella*–*Shigella* agar (SS, Criterion, Hardy diagnostic, California, United States) incubated at 37°C for 48 h.

### 2.8. Sensory Analysis

Sensory evaluation was performed following the method described by Lawless and Heymann [25]. A panel of 10 trained consumers (nine males and one female; age range: 20–30 years) from various ethnic groups (Sara, Tupuri, Kera, Mousgoum, N′gambaye, and Massa) participated. All panelists were familiar with the pendidam beverage from prior consumption and underwent a specific training session on the sensory attributes (color, odor, texture, acidity, and flavor) and the use of the nine‐point hedonic scale before the formal evaluation. They were fasting before the analysis, which began at 9:00 a.m., and each gave written informed consent. To avoid bias, samples were coded with three‐digit random numbers, presented in random order, and evaluated in individual booths under controlled lighting (approximately 800 lux, ~9 W LED lamps). No discussion was allowed among panelists. Each panelist received 30 mL of coded pendidam samples served at room temperature (25^°^ *C* ± 2^°^ *C*) in individual white cups. Drinking water (50 mL) was provided for mouth rinsing between samples. Sensory attributes, including color, odor, texture, acidity perception, flavor, and overall acceptability (OA), were assessed using a nine‐point hedonic scale ranging from 1 (“dislike extremely”) to 9 (“like extremely”).

### 2.9. Statistical Analysis

All data were recorded and coded using MS Excel 2013. Statistical analyses were performed using Statgraphics Centurion 16.1 software (Technologies Inc., Virginia, United States). Frequencies and percentages were calculated using descriptive analysis. Prior variance and correlation analyses, data normality and variances homogeneity were assessed using the Shapiro–Wilk and Levene tests, respectively. Since the variables and variances were normally distributed, one‐way analysis of variance (ANOVA) and Pearson′s correlation coefficient were used. One‐way ANOVA was applied to determine differences among samples. When significant effects were observed (*p* < 0.05), mean values were compared using Tukey′s honest significant difference (HSD) test. Results are presented as *m* *e* *a* *n* ± *s* *t* *a* *n* *d* *a* *r* *d* *d* *e* *v* *i* *a* *t* *i* *o* *n*. The association between selected sociodemographic parameters was assessed using the chi‐square (*χ* ^2^) correlation coefficient, with statistical significance considered for *p* value lower than 0.05 (*p* < 0.05). Pearson correlation coefficients (r) were used to examine relationships between analyzed parameters, and coefficients higher than 0.7 were considered indicative of a strong interaction. Principal component analysis (PCA) was performed to explore interrelationships among measured variables and across survey sites (observations). ANOVA, Association test, correlation analysis, and PCA were run using XLSTAT 2016.02.27444 software (Addinsoft, New York, United States).

## 3. Results

### 3.1. Sociodemographic Profile of Producers

Table 1 presents sociodemographic characteristics of pendidam producers. A survey of 80 producers across the four sites revealed that pendidam production is an exclusively female‐driven economic activity, deeply embedded in the pastoralist culture of the region. The producers were predominantly from the Peulh (Fulani) and Arab ethnic groups, with most being married (75%–90% across sites) and having an average age of 42.5 years. The vast majority (70%) of pendidam producers were between 30 and 50 years old. They were mainly from Cameroon (68.75%) and Chad (23.75%), and most of them (66.25%) reported having between 4 and 10 children. A large majority (90%) had no formal education, with rates reaching 100% in Maroua, 90% in Mora, and 85% in Kaélé and Mokolo. Conversely, experience in the trade was substantial, with over 90% of producers having more than 20 years of practice (92.5%), indicating that expertise is acquired through long‐term, informal apprenticeship rather than formal training.

**Table 1:** Sociodemographic profile of pendidam producers.

Criteria	Variables	Survey sites									
		Maroua		Kaélé		Mora	Mokolo				
		Fre	%	Fre	%	Fre	%	Fre	%	*N*	P (%)
Sex	Male	0	0	0	0	0	0	0	0	0	0
	Female	20	100	20	100	20	100	20	100	80	100

Marriage condition	Married	16	80	17	85	15	75	18	90	66	82.50
	Unmarried	4	20	3	15	3	15	1	5	11	13.75
	Divorcee	0	0	0	0	0	0	0	0	0	0
	Widowed	0	0	0	0	2	10	1	5	3	3.75

Number of children	None	2	10	1	5	1	5	2	10	6	7.50
	1–3	4	20	5	25	7	35	6	30	22	27.50
	4–6	6	30	9	45	7	35	9	45	31	38.75
	7–10	6	30	4	20	4	20	3	15	17	21.25
	> 10	2	10	1	5	1	5	0	0	4	5

Age (years)	< 30	4	20	2	10	3	15	4	20	13	16.25
	30–40	5	25	3	15	8	40	6	30	22	27.50
	40–50	8	40	9	45	7	35	10	50	34	42.50
	> 50	3	15	6	30	2	10	0	0	11	13.75

School level	Illiterate	20	100	17	85	18	90	17	85	72	90
	Primary	0	0	2	10	1	5	2	10	5	6.25
	Secondary	0	0	1	5	1	5	1	5	3	3.75
	University	0	0	0	0	0	0	0	0	0	0

Number of years of experience	< 5	0	0	0	0	0	0	0	0	0	0
	5–10	0	0	0	0	0	0	0	0	0	0
	11–15	2	10	1	5	1	5	0	0	4	5
	16–20	1	5	1	5	0	0	0	0	2	2.50
	> 20	17	85	18	90	19	95	20	100	74	92.50

Origin	Cameroon	14	70	13	65	12	60	16	80	55	68.75
	Chad	6	30	5	25	4	20	4	20	19	23.75
	Nigeria	0	0	0	0	2	10	0	0	2	2.50
	Niger	0	0	2	10	2	10	0	0	4	5

Abbreviations: %, percentage; Fre, fréquency; N, total frequency; P, average percentage.

As reported in Table 2, chi‐square tests revealed no significant association (*p* > 0.05) between survey site and most sociodemographic variables (marital status, age group, education level, number of children, and years of experience), indicating a homogeneous sociocultural profile of pendidam producers across the Far North Region of Cameroon. However, a significant association was revealed between survey site and producers′ origin (*χ* ^2^ = 19.86; *p* = 0.019), indicating geographical influence and cross‐border migration patterns. Significant internal sociodemographic associations were observed between age and number of children, marital status and number of children, and age and years of experience, highlighting expected demographic relationships within the study population. Strong association between age and years of experience (*χ* ^2^ = 41.62; *p* < 0.001) suggests that older producers have significantly more experience than younger ones. Great connection between age and number of children (*χ* ^2^ = 32.74; *p* = 0.001) confirms that the fertility pattern follows age structure, junior producers having less children than senior producers. Another significant association was found between marital condition and number of children (*χ* ^2^ = 28.15; *p* = 0.005), indicating that married producers had more children than single producers. Furthermore, there was a significant association between origin of producers and number of children (*χ* ^2^ = 23.17; *p* = 0.026), suggesting that family size also depends on cultural and socioeconomic background among pastoral populations. However, no significant association was revealed between the educational level and the years of experience of pendidam producers (*χ* ^2^ = 3.18; *p* = 0.785), confirming that experience in pendidam production is largely gained through traditional knowledge rather than formal schooling. This was reinforced by lack of association between age and education level (*χ* ^2^ = 6.27; *p* = 0.712), indicating uniformly low schooling across all age categories and highlighting the predominance of traditional knowledge in the pendidam production.

**Table 2:** Relationship between survey sites and sociodemographic determinants.

Parameters tested	df	*χ* ^2^‐value	*p*	Interpretation
Site × marital status	9	8.42	0.492	No association (similar distribution)
Site × children	12	10.87	0.540	No association (comparable patterns)
Site × age group	9	12.36	0.193	No association (moderate variation)
Site × education	6	5.21	0.517	No association (highly homogeneous)
Site × experience	9	7.44	0.593	No association (highly homogeneous)
Site × origin	9	19.86	0.019	Association (geographic migration influence)
Age × children	12	32.74	0.001	Association (natural demographic relationship)
Marital status × children	12	28.15	0.005	Association (family structure influence)
Origin × children	12	23.17	0.026	Association (family size depends on cultural background)
Age × experience	9	41.62	< 0.001	Association (senior producers more experienced)
Education × experience	6	3.18	0.785	No association (production experience not acquired through formal schooling)
Age × education	6	6.27	0.712	No association (limited access to formal education across all age categories)

*Note:* *p* value lower than 0.05 indicates a significant association.

Abbreviations: *χ* ^2^‐value, chi‐square value; df, degree of freedom.

### 3.2. Production and Selling Practices

**Figure 2:**
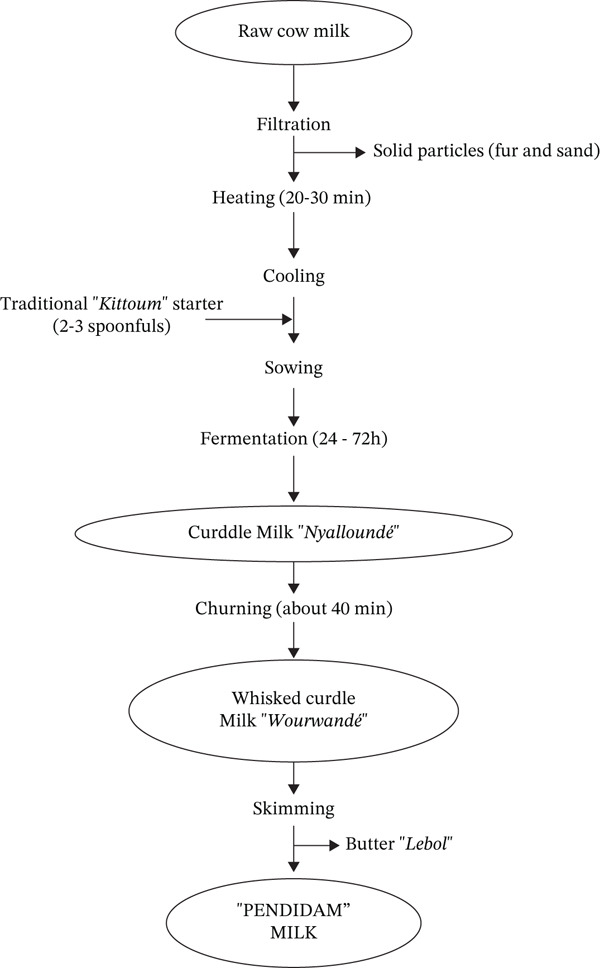
Traditional production of fermented pendidam milk. The flow chart has been elaborated by the authors based on field survey data collected in 2024.

**Figure 3:**
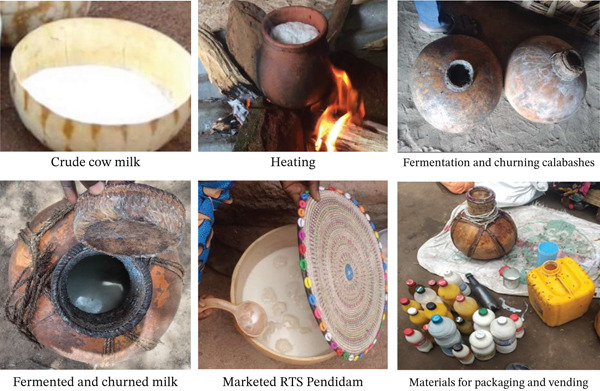
Pictorial of traditional processing and vending conditions of pendidam.

The production process, while artisanal, followed a set of unit operations, as detailed in Figure 2 and depicted in Figure 3. The process begins with the filtration of raw milk to remove impurities, followed by heating (Figure 3) at approximately 60°C–70°C for 20–30 min, a step that contributes to microbial reduction and improved product stability. After cooling to ambient temperature, the milk is inoculated with a small quantity of a previous successful batch, known locally as “kittoum” (2–3 spoonfuls), which acts as a natural starter culture. The inoculated milk is then incubated at ambient conditions, typically within a temperature range of 28°C–35°C, for 24–72 h, allowing for lactic acid fermentation, acidification, and coagulation, resulting in a curdled milk called “nyalloundé”. Subsequent churning for about 40 min produces a whisked curdled milk locally known as “wourwandé”. Skimming in a calabash separates the butter “lebol” from the fermented skimmed milk, which is the final product, pendidam.

**Figure 4:**
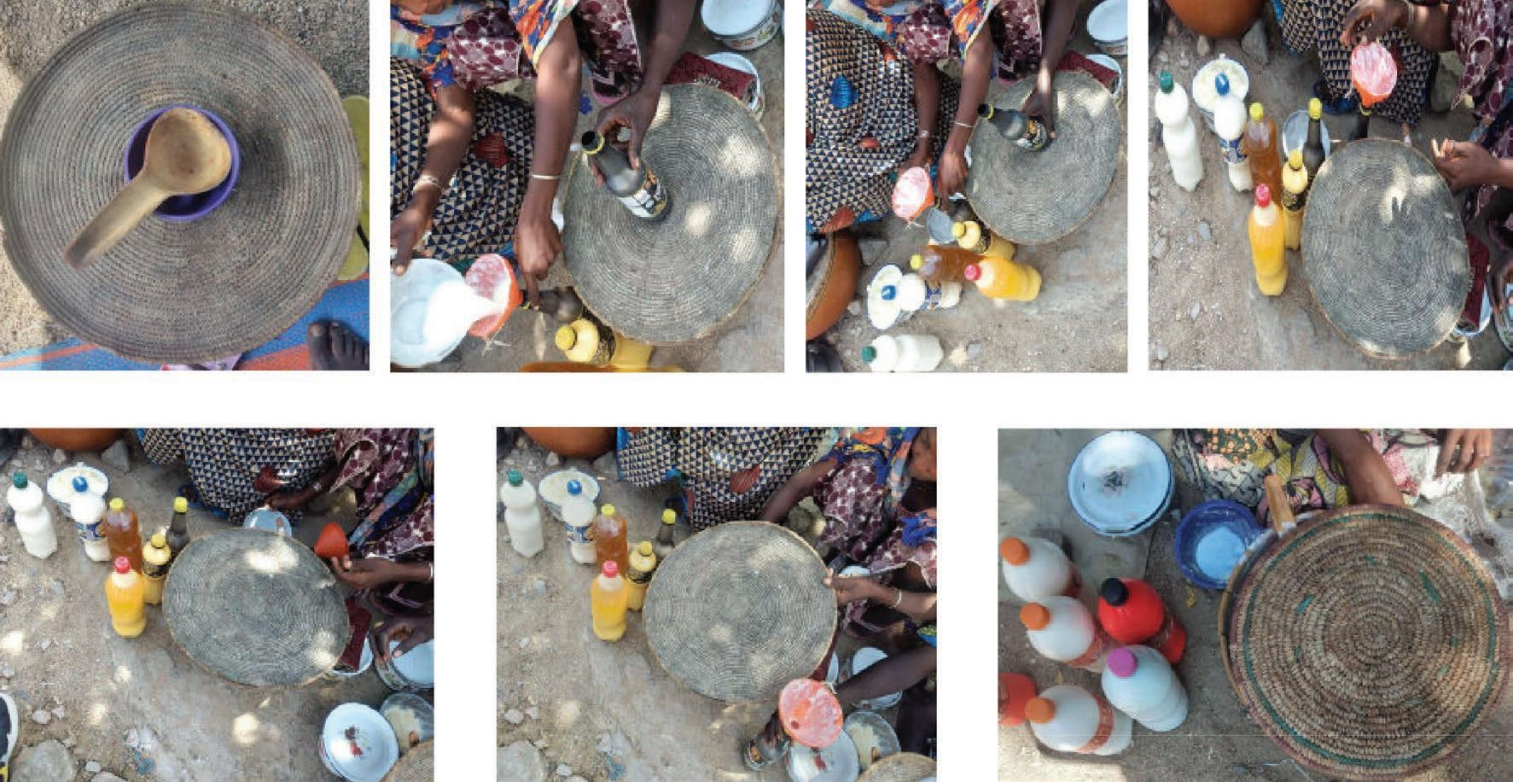
Serving tools, practices, and hygiene conditions during the marketing of pendidam milk.

Critical observations from the field survey highlighted several points of potential hygiene concern. The primary packaging materials were recycled plastic bottles (0.33 or 1 L) or traditional calabashes (Figure 3), which are often difficult to clean thoroughly. A common preservation technique involved adding whole grains of sorghum or beans (approximately 10 grains) directly into the storage containers, a practice believed by producers to extend shelf life through undefined antimicrobial properties. The water used for cleaning utensils was typically sourced from wells, boreholes, or seasonal “mayos” rivers, with no prior treatment. These factors, combined with the open‐air vending environments (as depicted in Figure 4) exposed to dust and insects, point to multiple avenues for postfermentation contamination. Pendidam is sold using measure tools in periodic markets (Figure 4). The product is measured either with a calabash ladle or with recycled plastic bottles (0.33 or 1 L). The average price ranges between US$0.40 and US$1.00. However, this price is variable and tends to be higher in urban areas than in rural zones, where the producers, who are often the sellers as well, typically reside.

### 3.3. Physicochemical Properties of Pendidam Milk

The physicochemical analysis confirmed pendidam as a highly acidic beverage (Table 3). The pH values across all samples were consistently low, ranging from 3.35 ± 0.14 in Kaélé to 3.66 ± 0.03 in Maroua, with no statistically significant variation (*p* > 0.05) between sites. This high acidity was quantitatively confirmed by TTA measurements, which vary from 0.68*%* ± 0.02*%* lactic acid in Maroua to 0.73*%* ± 0.02*%* in Mora. Significant differences (*p* < 0.05) were observed in other parameters. The soluble solids content, an indicator of dissolved sugars, was highest in samples from Maroua (3.06 ± 0.08°Brix) and lowest in Mokolo (2.30 ± 0.16°Brix). TDS and EC, reflecting mineral and ionic strength, showed similar trends with Maroua again having the highest values (1956.33 ± 23.09 ppm and 3948.33 ± 32.29 * μ*S/cm, respectively). The DM content, indicative of total solids, showed significant variation, with the highest percentage found in drink samples from Mora (6.27*%* ± 0.02*%*) and the lowest in Maroua (4.45*%* ± 0.06*%*).

**Table 3:** Physicochemical properties of pendidam milk.

Parameters	Sampling sites				Mean±SD
	Maroua	Mora	Mokolo	Kaélé	
pH	3.66 ± 0.03^b^	3.42 ± 0.07^a^	3.47 ± 0.05^a^	3.35 ± 0.14^a^	3.47 ± 0.14
Titratable acidity (%)	0.68 ± 0.02^a^	0.73 ± 0.02^b^	0.71 ± 0.04^ab^	0.72 ± 0.01^ab^	0.71 ± 0.03
Soluble solids (°Brix)	3.06 ± 0.08^a^	2.96 ± 0.08^a^	2.3 ± 0.16^a^	2.7 ± 0.10^a^	2 0.75 ± 0.32
Dissolved solids (ppm)	1956.33 ± 23.09^c^	1595.5 ± 127.35^a^	1807.17 ± 81.98^b^	1837.67 ± 14.52^b^	1799.17 ± 151.05
E. conductivity (*μ*S/cm)	3948.33 ± 32.29^d^	3271.83 ± 287.67^a^	3612.67 ± 163.95^b^	3741.0 ± 6.19^c^	3643.46 ± 295.08
Temperature (°C)	35.3 ± 0.17^d^	26.76 ± 0.55^b^	25.25 ± 0.27^a^	33.26 ± 0.13^c^	30.14 ± 4.35
Dry matter (%)	4.45 ± 0.06^a^	6.27 ± 0.02^c^	6.17 ± 0.31^c^	5.23 ± 0.02^b^	5.53 ± 0.80

*Note:* Mean values followed by the same letter (a, b, and c) are not statistically different (*p* > 0.05) in the same row according to ANOVA and HSD Tukey post hoc test.

### 3.4. Nutritional Composition of Pendidam Milk

Proximal analysis (Table 4) indicated that pendidam is nutritionally interesting, particularly in protein and sugars. The total proteins were significantly different (*p* < 0.05) across the sampling sites, with the highest content in samples from Kaélé (9.13 ± 0.09 g/L) and the lowest in Mora (7.84 ± 0.99 g/L).

**Table 4:** Proximate composition pendidam milk.

Parameters	Sampling sites				Mean±SD
	Maroua	Mora	Mokolo	Kaélé	
Soluble proteins (g/L)	8.79 ± 0.01^b^	7.84 ± 0.99^a^	9.05 ± 0.01^c^	9.13 ± 0.09^c^	8.70 ± 0.10
Amino acids (g/L)	6.99 ± 0.24^b^	5.72 ± 0.50^a^	7.77 ± 0.06^c^	5.61 ± 0.39^a^	6.52 ± 0.49
Soluble sugars (g/L)	7.68 ± 0.09^a^	8.17 ± 0.18^a^	8.87 ± 0.33^b^	8.01 ± 0.10^a^	8.18 ± 0.37
Reducing sugars (g/L)	5.92 ± 0.08^a^	7.91 ± 0.13^c^	7.72 ± 0.13^bc^	7.68 ± 0.09^b^	7.31 ± 0.23
Total ash (%)	0.39 ± 0.11^a^	0.34 ± 0.20^a^	0.48 ± 0.00^b^	0.48 ± 0.00^b^	0.43 ± 0.11

*Note:* Mean values followed by the same letter (a, b, and c) are not statistically different (*p* > 0.05) in the same row according to ANOVA and HSD Tukey post hoc test.

Free AA content, a product of protein breakdown during fermentation, also varied significantly, ranging from 5.60 ± 0.39 g/L in Kaélé to 7.77 ± 0.06 g/L in Mokolo. Total SS were highest in Mokolo (8.87 ± 0.33 g/L) and lowest in Maroua (7.68 ± 0.09 g/L). The content of RS was highest in Mora (7.91 ± 0.13 g/L), suggesting varying degrees of lactose hydrolysis during fermentation across sites. The ash content, representing total minerals, was relatively low overall (mean of 0.43%), with the highest value in pendidam milk from Mokolo (0.48%).

### 3.5. Microbiological Quality

Table 5 presents microbiological profile of pendidam milk. The microbiological assessment revealed a product of unsatisfactory hygienic quality, whereas the TAMF was high (5.80–5.88 Log_10_ CFU/mL) but within acceptable limits. The fungal flora (yeasts and molds) was remarkably high in all samples (5.48–5.78 Log_10_ CFU/mL), exceeding standard tolerance limits (< 5 Log_10_ CFU/mL). Similarly, mesophilic SFB were present above typical thresholds (4.44–4.90 Log_10_ CFU/mL). Staphylococci were detected in all samples at levels (2.79–2.99 Log_10_ CFU/mL) above safety limits, indicating potential hygiene failures during handling. Although total coliform (TC) counts were mostly below 3 Log_10_ CFU/mL, their presence indicates environmental contamination. RSC, associated with foodborne illness, were detected (2.56–3.13 Log_10_ CFU/mL). Positively, LAB were moderately present (6.80–7.01 Log_10_ CFU/mL), and no presumed *Salmonella* or *Shigella* spp. were detected.

**Table 5:** Microbiological profile of pendidam milk.

Microbiota (CFU/mL)	Sampling sites				Norms (log CFU/mL)	Mean±SD
	Maroua	Mora	Mokolo	Kaélé		
Total mesophilic bacteria	5.80 ± 0.01^a^	5.87 ± 0.01^bc^	5.86 ± 0.01^b^	5.88 ± 0.01^c^	< 6	5.85 ± 0.03
Total fungi	5.65 ± 0.02^c^	5.62 ± 0.01^b^	5.78 ± 0.01^d^	5.48 ± 0.01^a^	< 5	5.63 ± 0.10
Total coliforms	2.55 ± 0.67^a^	2.88 ± 0.04^bc^	2.92 ± 0.00^c^	2.86 ± 0.02^b^	< 3	2.80 ± 0.30
Fecal coliforms	0.90 ± 0.19^b^	No found	No found	0.60 ± 0.00^a^	< 2	0.37 ± 0.042
Staphylococci spp.	2.79 ± 0.01^a^	2.91 ± 0.00^b^	2.99 ± 0.01^d^	2.94 ± 0.02^c^	< 2	2.91 ± 0.08
Lactic acid bacteria	6.80 ± 0.02^a^	6.99 ± 0.00^c^	6.89 ± 0.00^b^	7.01 ± 0.00^d^	8–9	6.92 ± 0.09
Spore‐forming bacteria	4.56 ± 0.01^a^	4.44 ± 0.02^a^	4.90 ± 0.01^b^	4.56 ± 0.04^a^	< 4	4.61 ± 0.18
*Salmonella*/*Shigella*	Absent	Absent	Absent	Absent	Absence/25 g	—
Fecal streptococci	1.72 ± 0.06^a^	2.19 ± 0.00^c^	2.10 ± 0.03^b^	2.58 ± 0.05^d^	< 3	2.14 ± 0.32
Reducing sulfite Clostridia	2.56 ± 0.03^a^	3.13 ± 0.00^d^	3.04 ± 0.02^c^	2.58 ± 0.06^b^	< 5	2.83 ± 0.20

*Note:* Mean values followed by the same letter (a, b, c, and d) are not statistically different (*p* > 0.05) in the same row according ANOVA and HSD Tukey post hoc test.

### 3.6. Sensory Profile of Pendidam Milk

Sensory analysis indicated a differential appreciation linked to geographical origin as presented in Table 6. The highest scores for overall acceptability (OA) were given to samples from Mokolo (7.55 ± 0.72). Attributes such as color were best rated for samples from Mora and Kaélé (7.88 each), whereas flavor was highest for Mokolo (6.55 ± 1.01). The attribute with the lowest scores was acidity perception, particularly for samples from Maroua (4.66 ± 1.5).

**Table 6:** Hedonic scores of pendidam milk.

Sensory attributes	Sampling sites				Mean±SD
	Maroua	Mora	Mokolo	Kaélé	
Color	5.11 ± 2.14^a^	7.88 ± 0.92^b^	7.11 ± 1.36^b^	7.88 ± 0.60^b^	7.00 ± 1.75
Odor	5.88 ± 1.90^a^	6.88 ± 1.26^a^	7.22 ± 0.83^a^	6.22 ± 1.48^a^	6.55 ± 1.46
Texture	5.88 ± 2.02^a^	5.66 ± 2.0^a^	5.11 ± 1.90^a^	6.66 ± 1.11^a^	5.83 ± 1.46
Acidity perception	4.66 ± 1.50^a^	6.11 ± 1.45^a^	6.11 ± 1.16^a^	5.33 ± 2.06^a^	5.55 ± 1.62
Flavor	5.77 ± 1.98^a^	6.22 ± 1.39^a^	6.55 ± 1.01^a^	6.00 ± 1.00^a^	6.13 ± 1.37
Overall acceptability	5.77 ± 1.64^a^	7.00 ± 1.22^ab^	7.55 ± 0.72^b^	7.11 ± 1.53^ab^	6.86 ± 1.43

*Note:* Mean values followed by the same letter (a and b) are not statistically different (*p* > 0.05) in the same row according to ANOVA and HSD Tukey post hoc test.

### 3.7. Multivariate Analysis

**Figure 5:**
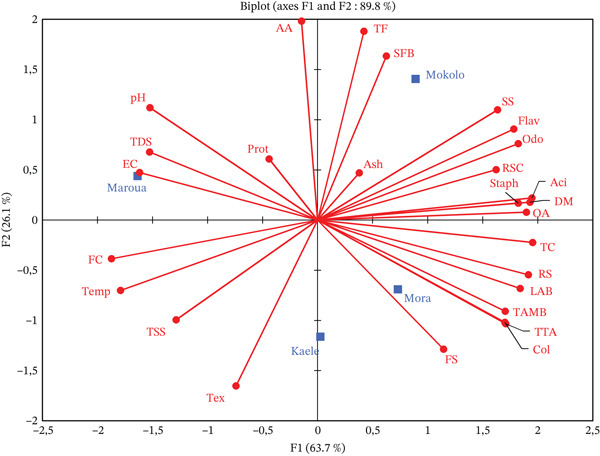
Biplot depicting interrelationship between pendidam milk properties and sampling locations. TTA, total titratable acidity; TSS, total soluble solids; TDS, total dissolved solids; EC, electrical conductivity; Temp, temperature; DM, dry matter; Prot, soluble proteins; AA, amino acids; SS, soluble sugars; RS, reducing sugars; TAMB, total mesophilic bacteria; TF, total fungi; TC, total coliforms; FC, fecal coliforms; Staph, staphylococci; LAB, lactic acid bacteria; SFB, spore‐forming bacteria; FS, fecal streptococci; RSC, reducing sulfite clostridia; Col, color; Tex, texture; Odo, odor; Aci, acidity perception; Flav, flavor; OA, overall acceptability.

As shown in Figure 5, the PCA biplot, accounting for a high cumulative variance of 82.96% (*F*1 = 59.44*%* and *F*2 = 23.52*%*), provides a robust and comprehensive representation of the relationships among physicochemical, microbiological, and sensory attributes of pendidam milk while it clearly discriminates samples according to their geographical origin.

The first principal component (F1), which explains the largest proportion of variability, can be interpreted as a global fermentation, compositional, contamination, and acceptability gradient. It represents the dominant factor opposing highly fermented, microbiologically active, and sensory appreciated samples to less transformed and physicochemical driven samples. On the positive side of F1, variables such as total mesophilic aerobic bacteria (TAMB), TC, LAB, FS, RSC, and Staphylococci (Staph) are positively aligned with TTA, SS, RS, and DM, as well as key sensory attributes including OA, perceived acidity (Aci), flavor (Flav), odor (Odo), and color (Col). This clustering indicates that samples with higher sugar contents and microbial loads are also those exhibiting more advanced fermentation, greater acidification, and enhanced sensory appreciation. Conversely, the negative side of the F1 axis is characterized by higher pH, EC, TDS, temperature (Temp), total soluble solids (TSS), and FC, reflecting beverages that are low fermented, less acidic, and facing fecal contamination likely from raw milk.

The second principal component (F2) further refines this differentiation by separating samples according to the nutritional richness and microbial contamination. The positive side of F2 is associated with nutritional features such as AA, soluble proteins (Prot), and ash, as well as microbiota including TF and SFB. In contrast, the negative side of F2 is dominated by variables such as FS and texture (Tex).

The projection of sampling locations onto the biplot demonstrates a clear geographical structuring of pendidam milk characteristics likely reflecting the combined effect of slight differences in processing practices, fermentation duration, variability in raw milk characteristics, and local environmental and hygiene conditions. The samples from Maroua are positioned on the negative side of F1 and are closely associated with pH, EC, and TDS, indicating that samples from this location are less fermented, exhibit lower microbial activity, and retain more of their initial physicochemical properties. This suggests either shorter fermentation times or relatively improved handling practices that limit microbial contamination and proliferation. In contrast, Mora samples are located in the lower quadrant delimited by the positive side of F1 and negative side of F2, aligning strongly with LAB, TAMB, RS, TC, FS, and TTA. This positioning indicates intense lactic fermentation accompanied by high microbial loads, including indicators of fecal contamination, reflecting both advanced fermentation and potential hygiene deficiencies during production or storage. Samples from Mokolo located in the upper quadrant formed by the positive regions of both F1 and F2, associate with sensory attributes including OA, Flav, Odo, Aci, microbial features such as Staph, RSC, SFB, and TF. Given that samples from Mokolo are closely related to F2, they are also associated with higher AA and ash contents. This indicates that beverage from this location is sensory superior, higher prevalence of undesirable microbial indicators, and interesting nutritional properties. Kaele samples, located near the lower central region of the biplot, associate with the dominant variables (texture and FS) in the negative part of F2, indicating an intermediate profile with moderate fermentation and less pronounced microbiological or sensory characteristics.

### 3.8. Correlation Analysis

Examining the complete correlation matrix (Table 7) reveals complex interrelationships among the physicochemical, proximate, microbiological, and sensory characteristics of pendidam milk. OA demonstrates extraordinarily strong positive correlations with staphylococci (*r* = 0.99), TC (*r* = 0.98), and RS (*r* = 0.93), while also showing very strong positive connections with DM (*r* = 0.84) and SS (*r* = 0.85). This pattern indicates that the samples perceived as most acceptable by the panelists were precisely those with higher concentrations of fermentable sugars, greater solids content, and paradoxically, higher microbial loads. Furthermore, OA was inversely correlated with pH (*r* = −0.80), suggesting that panelists preferred more acidic samples.

**Table 7:** Pearson coefficients matrix between physicochemical, proximate, microbiological, and sensory characteristics of pendidam.

Parameters	pH	TTA	TSS	TDS	E.C	Tem	DM.	Prot.	AA	SS	RS	Ash	TMB	TF	TC	FC	Staph	LAB	SFB	FS	RSC	Col	Odo	Tex	Aci	Flav	OA
pH	1.00	**−0.92**	0.40	0.59	0.57	0.40	−0.60	0.04	0.57	−0.37	**−0.91**	−0.32	**−0.99**	0.45	**−0.87**	0.12	**−0.78**	**−0.96**	−0.25	**−0.84**	−0.12	**−0.89**	−0.42	0.11	**−0.81**	−0.45	**−0.80**
TTA	**−0.92**	1.00	−0.25	**−0.86**	**−0.85**	−0.60	**0.79**	−0.41	−0.58	0.40	**0.95**	−0.02	**0.94**	−0.29	**0.88**	0.15	**0.71**	**0.94**	0.33	**0.89**	0.29	**0.80**	0.27	−0.18	**0.85**	0.35	**0.77**
TSS	0.40	−0.25	1.00	0.00	0.09	0.59	−0.52	−0.55	−0.51	**−0.90**	−0.54	**−0.79**	−0.47	−0.45	0.30	0.30	−0.32	−0.49	0.29	−0.33	0.44	−0.45	−0.53	−0.15	−0.31	−0.47	**−0.81**
TDS	0.59	**−0.86**	0.00	1.00	**0.99**	**0.72**	**−0.84**	**0.78**	0.45	−0.34	**−0.77**	0.46	−0.66	0.01	0.04	0.36	0.12	−0.27	0.56	−0.15	0.51	−0.24	−0.28	−0.08	−0.19	−0.36	−0.54
E.C	0.57	**−0.85**	0.09	**0.99**	1.00	**0.79**	**−0.89**	**0.75**	0.35	−0.44	**−0.79**	0.42	−0.66	−0.09	−0.08	0.36	−0.16	−0.31	0.56	−0.19	0.51	−0.28	−0.30	−0.07	−0.23	−0.38	−0.59
Tem	0.40	−0.60	0.59	**0.72**	**0.79**	1.00	**−0.96**	0.35	−0.25	**−0.88**	**−0.74**	0.01	−0.53	−0.59	**−0.79**	−0.51	**−0.74**	−0.30	**−0.74**	−0.60	**−0.84**	0.20	0.55	0.57	0.19	0.38	**−0.78**
DM	−0.60	**0.79**	−0.52	**−0.84**	**−0.89**	**−0.96**	1.00	−0.42	−0.01	**0.79**	**0.87**	−0.04	**0.71**	0.35	**0.89**	0.41	**0.78**	0.54	0.60	**0.80**	**0.79**	0.01	−0.41	−0.47	0.01	−0.22	**0.84**
Prot.	0.04	−0.41	−0.55	**0.78**	**0.75**	0.35	−0.42	1.00	0.42	0.15	−0.20	**0.91**	−0.08	−0.01	0.08	−0.05	0.20	−0.25	0.17	−0.09	0.02	−0.19	−0.50	−0.54	−0.34	−0.44	0.10
AA	0.57	−0.58	−0.51	0.45	0.35	−0.25	−0.01	0.42	1.00	0.51	−0.33	0.32	−0.48	**0.89**	−0.16	**0.71**	0.06	**−0.76**	0.67	−0.38	0.56	−0.59	−0.63	0.42	−0.69	−0.62	0.00
SS	−0.37	0.40	**−0.90**	−0.34	−0.44	**−0.88**	**0.79**	0.15	0.51	1.00	0.65	0.45	0.49	0.65	**0.77**	0.68	**0.86**	0.16	**0.84**	0.63	**0.93**	−0.05	−0.44	−0.56	−0.09	−0.29	**0.85**
RS	**−0.91**	**0.95**	−0.54	**−0.77**	**−0.79**	**−0.74**	**0.87**	−0.20	−0.33	0.65	1.00	−0.11	**0.85**	0.12	**0.98**	0.09	**0.89**	**0.85**	0.31	**0.95**	0.29	**0.73**	0.19	−0.18	0.78	0.30	**0.93**
Ash	−0.32	−0.02	**−0.79**	0.46	0.42	0.01	−0.04	**0.91**	0.32	0.45	−0.11	1.00	0.17	0.11	0.35	0.39	0.47	0.13	0.55	0.28	0.59	0.07	−0.13	−0.34	0.06	−0.05	0.49
TMB	**−0.99**	**0.94**	−0.47	−0.66	−0.66	−0.53	**0.71**	−0.08	−0.48	0.49	**0.85**	0.17	1.00	−0.34	**0.92**	−0.08	**0.75**	**0.80**	0.27	**0.87**	0.29	0.69	0.16	−0.25	0.70	0.26	**0.87**
TF	0.45	−0.29	−0.45	−0.09	−0.09	−0.59	0.35	−0.01	**0.89**	0.65	0.12	0.11	−0.34	1.00	0.05	**0.88**	0.16	−0.59	**0.80**	−0.17	**0.76**	−0.53	**−0.70**	−0.52	−0.58	−0.63	0.15
TC	**−0.87**	**0.88**	0.30	0.04	−0.08	**−0.79**	**0.89**	0.08	−0.16	**0.77**	**0.98**	0.35	**0.92**	0.05	1.00	0.21	**0.95**	**0.75**	0.41	**0.97**	0.42	0.65	0.09	−0.26	**0.72**	0.24	**0.98**
FC	0.12	0.15	0.30	0.36	0.36	−0.51	0.41	−0.05	**0.71**	0.68	0.09	0.39	−0.08	**0.88**	0.21	1.00	0.33	−0.25	**0.92**	0.11	**0.83**	−0.37	−0.49	−0.37	−0.26	−0.35	0.32
Staph	**−0.78**	**0.71**	−0.32	0.12	−0.16	**−0.74**	**0.78**	0.20	0.06	**0.86**	**0.89**	0.47	**0.75**	0.16	**0.95**	0.33	1.00	0.59	0.57	**0.90**	0.62	0.48	−0.09	−0.44	0.53	0.06	**0.99**
LAB	−**0.96**	**0.94**	−0.49	−0.27	−0.31	−0.30	0.54	−0.25	**−0.76**	0.16	**0.85**	0.13	**0.80**	−0.59	**0.75**	−0.25	0.59	1.00	−0.09	**0.84**	0.01	**0.80**	0.44	0.03	0**.83**	0.47	0.64
SFB	−0.25	0.33	0.29	0.56	0.56	**−0.74**	0.60	0.17	0.67	**0.84**	0.31	0.55	0.27	**0.80**	0.41	**0.92**	0.57	−0.09	1.00	0.24	**0.89**	−0.08	−0.40	−0.36	−0.07	−0.20	0.48
FS	**−0.84**	**0.89**	−0.33	−0.15	−0.19	−0.60	**0.80**	−0.09	−0.38	0.63	**0.95**	0.28	**0.87**	−0.17	**0.97**	0.11	**0.90**	**0.84**	0.24	1.00	0.33	0.65	0.14	−0.23	**0.73**	0.28	0.68
RSC	−0.12	0.29	0.44	0.51	0.51	**−0.84**	**0.79**	0.02	0.56	**0.93**	0.29	0.59	0.29	**0.76**	0.42	**0.83**	0.62	0.01	**0.89**	0.33	1.00	−0.12	−0.46	−0.50	−0.08	−0.28	0.61
Col	**−0.89**	**0.80**	−0.45	−0.24	−0.28	0.20	0.01	−0.19	−0.59	−0.05	**0.73**	0.07	0.69	−0.53	0.65	−0.37	0.48	**0.80**	−0.08	0.65	−0.12	1.00	**0.77**	0.36	**0.86**	**0.78**	**0.83**
Odo	−0.42	0.27	−0.53	−0.28	−0.30	0.55	−0.41	−0.50	−0.63	−0.44	0.19	−0.13	0.16	**−0.70**	0.09	−0.49	−0.09	0.44	−0.40	0.14	−0.46	**0.77**	1.00	**0.78**	0.65	**0.93**	**0.83**
Tex	0.11	−0.18	−0.15	−0.08	−0.07	0.57	−0.47	−0.54	0.42	−0.56	−0.18	−0.34	−0.25	−0.52	−0.26	−0.37	−0.44	0.03	−0.36	−0.23	−0.50	0.36	**0.78**	1.00	0.51	**0.79**	−0.25
Aci	**−0.81**	**0.85**	−0.31	−0.19	−0.23	0.19	0.01	−0.34	−0.69	0.09	**0.78**	0.06	0.70	−0.58	**0.72**	−0.26	0.53	**0.83**	−0.07	**0.73**	−0.08	**0.86**	0.65	0.51	1.00	**0.76**	0.60
Flav	−0.45	0.35	−0.47	−0.36	−0.38	0.38	0.22	−0.44	−0.62	−0.29	**0**.30	−0.05	0.26	−0.63	0.24	0.35	0.06	0.47	−0.20	0.28	−0.28	**0.78**	**0.93**	**0.79**	**0.76**	1.00	**0.87**
OA.	**−0.80**	**0.77**	**−0.81**	−0.54	−0.59	**−0.78**	**0.84**	0.10	0.00	**0.85**	**0.93**	0.49	**0.87**	0.15	**0.98**	0.32	**0.99**	0.64	0.48	0.68	0.61	**0.83**	**0.83**	−0.25	0.60	**0.87**	1.00

*Note:* Coefficients statistically significant (*p* < 0.05) are highlighted in bold.

Abbreviations: AA, amino acids; Aci, acidity perception; Col, color; DM, dry matter; EC, electrical conductivity; FC, fecal coliforms; Flav, flavor; FS, fecal streptococci; LAB, lactic acid bacteria; OA, overall acceptability; Odo, odor; Prot, soluble proteins; RS, reducing sugars; RSC, reducing sulfite clostridia; SFB, spore−forming bacteria; SS, soluble sugars; Staph, staphylococci; TC, total coliforms; TDS, total dissolved solids; Tem, temperature; Tex, texture; TF, total fungi; TMB, total mesophilic bacteria; TSS, total soluble solids; TTA, total titratable acidity.

Among the sensory attributes, notable patterns emerge. Color shows positive relationships with acidity perception (*r* = 0.86), flavor (*r* = 0.78), and odor (*r* = 0.77), while being negatively correlated with pH (*r* = −0.89) and positively with TTA (*r* = 0.80). This indicates that the characteristic color of pendidam milk is associated with the degree of fermentation. Odor and flavor exhibit a strong mutual correlation (*r* = 0.93), showing that these two sensory attributes are perceived nearly identically by panelists. Both odor and flavor show strong positive correlations with color (*r* = 0.77 and *r* = 0.78, respectively), texture (*r* = 0.78 and *r* = 0.79, respectively), and OA (*r* = 0.83 and *r* = 0.87, respectively). This finding suggests that odor and flavor are internally consistent; they drive OA in the same way that acidity does. Acidity perception, the sensory rating of sourness, correlates strongly with color (*r* = 0.86) and flavor (*r* = 0.76), as well as with TTA (*r* = 0.85) and LAB (*r* = 0.83), confirming that panelists accurately perceived the actual chemical acidity of the samples.

The acidity parameters reveal a tightly coordinated system. pH and TTA are almost perfectly inversely correlated (*r* = −0.92), as expected. LAB show a strong negative correlation with pH (*r* = −0.96) and a correspondingly strong positive correlation with TTA (*r* = 0.94), confirming their primary role in acidification through sugar fermentation. This is further supported by the strong positive correlation between LAB and RS (*r* = 0.85) and the inverse relationship between RS and pH (*r* = −0.91).

The microbiological profile reveals extensive intercorrelations. TC shows near‐perfect correlations with RS (*r* = 0.98), FS (*r* = 0.97), staphylococci (*r* = 0.95), and total mesophilic bacteria (*r* = 0.92). Staphylococci demonstrate similarly strong associations with RS (*r* = 0.89), TC (*r* = 0.95), FS (*r* = 0.90), and overall acceptability (*r* = 0.99). These tight clusters suggest that these microbial groups co‐occur in the same samples, likely reflecting common contamination sources or growth conditions.

Temperature exhibits strong negative correlations with DM (*r* = −0.96), SS (*r* = −0.88), RS (*r* = −0.74), and multiple microbial groups including reducing sulfite clostridia (*r* = −0.84), SFB (*r* = −0.74), and TC (*r* = −0.79). This indicates that cooler storage or production temperatures preserve both solid concentration and microbial viability. DM content, as expected, correlates strongly with nearly all components that contribute to total solids, including RS (*r* = 0.87), SS (*r* = 0.79), TC (*r* = 0.89), and FS (*r* = 0.80). EC and dissolved solids are nearly perfectly correlated with each other (*r* = 0.99), as expected, but show relatively weak relationships with most other variables, suggesting that ionic content is largely independent of the fermentation and microbial processes driving other parameters.

## 4. Discussion

This study provides important baseline data on pendidam, a traditional fermented milk that constitutes a cornerstone of the diet and a key contributor to the local economy in the Far North Region of Cameroon. The findings revealed a clear dichotomy in pendidam milk. The beverage is a nutritionally valuable and culturally embedded food whose production, consumption, and marketability are shadowed, however, by significant and systemic hygienic shortcomings.

The exclusive involvement of women producers with limited formal education but extensive practical experience confirms the gendered and empirical nature of this indigenous knowledge system, aligning with patterns observed across West Africa where dairy transformation is often a female domain [3]. Although this empirical knowledge ensures the continuity of production practices, it also presents a barrier to the adoption of standardized hygienic procedures. The lack of association between education level and production experience suggests that practical, experiential learning, rather than formal training, is the primary mechanism for knowledge transmission. This orally transmitted system has resulted in an apparent uniformity of processing steps across the four study locations. However, this reliability also perpetuates systemic routes of contamination, including the use of recycled containers, untreated water, spontaneous fermentation, and ambient storage under unhygienic vending conditions. These risk factors are not unique to Cameroon; similar challenges have been reported for traditional fermented milks in neighboring countries, including products from Chad [17], suggesting that these are structural, systemic issues rather than product‐specific anomalies. Furthermore, differences in processing compared with pendidam produced in the Adamawa Region of Cameroon [16] highlight the influence of cultural and geographical factors on traditional food technologies [26]. The use of unique fermentation process in the Far North rather than a multistage process as reported in product from Adamawa may affect microbial succession and acidification kinetics, potentially explaining variations in physicochemical and microbiological characteristics between both northern regions. Such heterogeneity underscores the difficulty of generalizing safety or nutritional assessments across culturally distinct production systems bearing the same product name.

The physicochemical results confirm that pendidam is a strongly acidified fermented milk, with pH and TTA values typical of spontaneous lactic fermentations. These parameters reflect active lactose conversion by LAB, which are expected to dominate in traditional dairy fermentations [5, 15, 27]. This acidic environment is a fundamental characteristic that underpins both the nutritional potential of products and its inherent, though demonstrably insufficient, preservation mechanism. However, the observed variability in soluble solids, dissolved solids, and conductivity among samples suggests differences in raw milk composition, fermentation intensity, or the extent of lactose hydrolysis [28, 29]. Factors such as animal breed, feeding regime, lactation stage, and seasonal forage availability likely contribute to this variability [30]. Moreover, the high conductivity values may indicate enhanced mineral solubilization under acidic conditions, particularly of calcium and phosphate complexes [31].

Nutritionally, pendidam emerges as a potential source of proteins and essential AA. The levels of soluble free AA indicate extensive proteolysis during fermentation, likely driven by the diverse microbial consortium. This activity may enhance protein digestibility and increase the bioavailability of essential AA [32]. Similarly, the presence of SS and RS suggests that while lactose is being converted, residual sugars remain and other carbohydrates point to the production of microbial metabolites, such as exopolysaccharides that may influence viscosity and mouthfeel [33, 34]. Although these characteristics support the classification of pendidam as a nutritional beverage, they also demonstrate the absence of process standardization. Critically, the same nutrients that confer nutritional benefits can also support the growth of opportunistic microbial contaminants, particularly under conditions of postfermentation contamination, thereby reducing shelf stability and increasing risk.

The PCA provides a powerful framework for synthesizing these complex interactions, clearly delineating the primary axes of variation that define pendidam quality and safety. The first and most significant axis (PC1) represents a fundamental quality gradient, contrasting highly fermented, microbiologically active, and sensory appreciated samples to less transformed and physicochemical driven samples. Strikingly, this axis reveals a critical trade‐off revealing that the samples with high LAB counts and elevated consumer acceptability are the same ones that, worryingly, show high counts of hygienic indicator microorganisms such as TC and Staph. This finding is the central paradox of pendidam. This apparent paradox between high sensory acceptability and elevated microbial loads in traditional fermented milk products such as pendidam can be explained by the functional role of microorganisms in shaping product quality. In these artisanal systems, the same microbial groups that contribute to high counts, particularly LAB and associated fermentative flora, are responsible for producing organic acids, volatile compounds, and exopolysaccharides, which enhance acidity, aroma, and texture. The positive correlations observed between acceptability and sugar or DM content further indicate that intense fermentation yields a balanced sweet–acid profile, which is locally preferred. Moreover, hygiene indicator microorganisms (TC, FC, and FS) may reflect in this context overall microbial abundance and an advanced fermentation stage rather than product spoilage. Furthermore, consumers accustomed to traditionally fermented products often associate stronger flavors and thicker consistency, linked to higher microbial activity, with superior quality, reflecting a cultural adaptation of sensory expectations. However, this sensory‐driven acceptability does not negate food safety concerns. The presence of high levels of hygiene indicators still signals potential health risks. Therefore, this finding underscores the need to improve hygienic practices or introduce controlled fermentation strategies that preserve desirable sensory properties while reducing microbiological hazards. Under current unhygienic practices, the fermentation process that creates desirable sensory and nutritional properties cannot be disentangled from the introduction of contaminants. This suggests either that these indicator microorganisms are not reliable markers of spoilage in this specific ecological context or, more worryingly, that consumer preferences for acidity and texture are directly correlated with other factors such as sugar content that also favor microbial growth [35, 36]. Mokolo emerges as the site associated with the most favorable overall profile, and Maroua as less fermented retaining more of its initial physicochemical properties. The second axis (PC2) further differentiates samples based on specific contamination patterns and nutritional richness. Mora emerges as the most distinct site, clustering separately due to high loads of TC and FS, pointing to site‐specific contamination sources, such as fecally contaminated water or milk.

The microbiological profile, viewed through the lens of the PCA, raises significant and specific food safety concerns based on relevant microbial criteria from the International Commission on Microbiological Specifications for Foods [37]. The high counts of TC, FS, and Staph, particularly their association with highly acceptable samples along PC1, demonstrate that hygienic inadequacies are pervasive and deeply integrated into the production and handling chain. The detection of RSC, especially in Mokolo, indicates possible environmental contamination from soil, water, or improperly cleaned equipment, whereas the high yeast and mold loads point to airborne contamination during storage and marketing [38]. The persistence of these microorganisms despite the low pH is particularly concerning. It suggests the presence of acid‐tolerant strains [39], uneven acid distribution within the product forming pH microenvironments that protect pathogens [40], or simply that contamination occurs postfermentation, bypassing the acidic hurdle [41]. This indicates that relying on pH as a critical control point is insufficient, and interventions must target contamination before and after fermentation begins. Although the absence of *Salmonella* and *Shigella* is encouraging, it does not eliminate the risk of foodborne illness, particularly given the presence of potential toxin‐producing staphylococci. The inhibitory effect of acidic conditions and competitive LAB flora against these specific enteric pathogens (*Salmonella* and *Shigella*) may explain their nondetection [42], but it offers no guarantee against others such as FC. The observed heterogeneity in FC detection across milk samples likely reflects differences in hygienic practices and contamination sources along the production and distribution chain. Samples that tested negative for FC may have benefited from relatively better handling conditions, such as the use of cleaner utensils, safer water sources, or reduced postfermentation contamination. In contrast, FC contaminated samples (below standard safety thresholds of 2 Log_10_) probably resulted from direct or indirect fecal contamination through water, handlers, or environmental exposure during artisanal processing and marketing. Importantly, the intermittent nature of detection suggests that FC contamination is not intrinsic to the fermentation process itself but rather occurs sporadically, depending on local hygiene conditions. Differences in mesophilic aerobic counts between sampling locations can be attributed to multiple factors, including variations in raw milk quality, ambient temperature, fermentation time and conditions, and postprocessing handling practices. Although the acidic nature of pendidam (pH lower than 4) generally inhibits the growth of many mesophilic bacteria, it does not entirely eliminate them. Some acid‐tolerant or acid‐adapted mesophilic populations may persist, especially when acidification is slow or uneven, or when postfermentation contamination occurs. Consequently, the observed mesophilic counts likely reflect a balance between microbial inhibition and survival or recontamination of adapted strains, and this balance may vary across locations due to differing environmental and hygienic conditions.

The sensory evaluation reveals a critical public health blind spot that is perfectly illustrated by the aforementioned PCA. The fact that highly appreciated samples are those with elevated contamination levels (along PC1) indicates that sensory attributes are not reliable and are in fact potentially deceptive food safety indicators. This phenomenon is common in traditional fermented beverages [43–45], where desirable organoleptic characteristics may coexist with poor hygienic quality [46]. The danger is clear given that the consumers are actively selecting the products that pose the highest potential risk. The strong associations among RS, acidity, LAB, and overall acceptability in the correlation structure point to fermentation intensity as a key driver of consumer preference. Panelists favor samples that have undergone more extensive acidification and sugar conversion, unwittingly preferring a process that, in the absence of hygienic practices, is linked to higher contaminant loads.

Although this study provides a robust baseline, it is limited by its cross‐sectional design. Future research should employ longitudinal sampling to capture seasonal variations in pendidam milk composition and microbial ecology, and use metagenomics to identify the microbial species present, including potential pathogens and their functional genes. Another limitation of our work was the male imbalance of the sensory panel. Therefore, we plan in our future sensory evaluations to adopt a stratified recruitment strategy to ensure a more gender‐balanced panel, by scheduling sessions at different times of the day or by providing alternative accommodations to facilitate female participation.

## 5. Conclusion

This work reports a comprehensive characterization of pendidam, underscoring its dual nature as a valuable traditional food and a product of serious hygienic concern. The multivariate analysis confirms that pendidam is a complex biological system where physicochemical parameters, microbial communities, and sensory properties are deeply and paradoxically often connected. The documented production process, while remarkably rooted in indigenous knowledge, incorporates multiple critical control points where contamination occurs, primarily due to the use of nonpotable water, unhygienic utensils, empirical fermentation, inadequate storage, and unsanitary vending conditions. These practices directly explain the unacceptable microbial loads of hygiene indicators (coliforms and staphylococci) and hazardous microorganisms (RSC and fungi) found in the analyzed samples. Despite this, the appealing sensory profile and nutritional composition of fermented pendidam milk explain its enduring popularity and relatively high consumer acceptability. The findings clearly indicate that the empirical nature of the production technology of pendidam is the primary limitation to its safety and scalability. Therefore, the valorization of pendidam hinges on targeted technological improvements that respect its cultural essence while integrating basic food safety principles. Strategic recommendations include implementing low‐cost sanitization protocols for equipment and packaging; developing and use of a well‐characterized, multistrain starter culture to ensure a controlled and safe fermentation; providing formal and practical hygiene education for producers; and improving sanitary conditions during marketing of the beverage. Such interventions codesigned with the women producers would mitigate public health risks and standardize quality, transforming pendidam milk from a locally consumed and high‐risk product into a safe beverage with potential for broader market appeal and socioeconomic impact.

## Author Contributions

James Ronald Bayoï: conceptualization, resources, visualization, supervision, writing review. Simplice Noubasra: formal and data analysis, resources, writing the first draft.

## Funding

No funding was received for this manuscript.

## Ethics Statement

All participants who gave us their consent before the analysis approved the sensory analysis. This part of the study was carried out in accordance with the Code of Ethics of the World Medical Association for experiments involving humans.

## Conflicts of Interest

The authors declare no conflicts of interest.

## Data Availability

All data used to support the findings of this study are included in this article.
